# CAMKII-conditional deletion of histone deacetylase 2 potentiates acute methamphetamine-induced expression of immediate early genes in the mouse nucleus accumbens

**DOI:** 10.1038/srep13396

**Published:** 2015-08-24

**Authors:** Oscar V. Torres, Michael T. McCoy, Bruce Ladenheim, Subramaniam Jayanthi, Christie Brannock, Ingrid Tulloch, Irina N. Krasnova, Jean Lud Cadet

**Affiliations:** 1 Molecular Neuropsychiatry Research Branch, DHHS/NIH/NIDA Intramural Research Program, 251 Bayview Boulevard, Baltimore, MD 21224; 2 Department of Psychology, Stevenson University, Stevenson, MD 21283

## Abstract

Methamphetamine (METH) produces increases in the expression of immediate early genes (IEGs) and of histone deacetylase 2 (HDAC2) in the rat nucleus accumbens (NAc). Here, we tested whether HDAC2 deletion influenced the effects of METH on IEG expression in the NAc. Microarray analyses showed no baseline differences in IEG expression between wild-type (WT) and HDAC2 knockout (KO) mice. Quantitative-PCR analysis shows that an acute METH injection produced time-dependent increases in mRNA levels of several IEGs in both genotypes. Interestingly, HDAC2KO mice displayed greater METH-induced increases in *Egr*1 and *Egr*2 mRNA levels measured at one hour post-injection. The levels of *Fosb*, *Fra*2, *Egr1*, and *Egr3* mRNAs stayed elevated in the HDAC2KO mice 2 hours after the METH injection whereas these mRNAs had normalized in the WT mice. In WT mice, METH caused increased HDAC2 recruitment to the promoters some IEGs at 2 hours post injection. METH-induced prolonged increases in *Fosb*, *Fra2*, *Egr1*, and *Egr3* mRNA levels in HDAC2KO mice were associated with increased enrichment of phosphorylated CREB (pCREB) on the promoters of these genes. Based on our observations, we hypothesize that HDAC2 may regulate the expression of these genes, in part, by prolonging the actions of pCREB in the mouse NAc.

Methamphetamine (METH) is an addictive psychostimulant with an estimated 25 million users worldwide^1^. In humans, acute METH produces a sense of euphoria and increased energy^2^. In contrast, chronic METH use is associated with negative consequences including neurocognitive deficits^3^,^4^. These adverse consequences are secondary to drug-induced altered brain function and structures^5^,^6^,^7^. METH administration increases locomotor activity, produces conditioned place preferences, and is self-administered^8^,^9^,^10^ by rodents. These behaviors have been attributed to METH-induced release of dopamine (DA) in reward-associated brain regions including the nucleus accumbens (NAc)^11^,^12^. METH administration is also accompanied by changes in gene expression in the rodent brain^13^,^14^. Specifically, acute METH injections increase the expression of several immediate early genes (IEGs) including members of the *Fos*, *Jun*, *Egr*, and of the nuclear receptor subfamily 4, group A (*Nr4a*) families of transcription factors (TFs) in the NAc and dorsal striatum^13^,^15^,^16^,^17^.

Gene transcription is regulated by epigenetic phenomena that include chromatin modifications, post-translational histone alterations, and changes in the binding of transcription factors (TFs) at gene promoters^18^. In eukaryotic cells, DNA exists as chromatin that is composed of 4 core histones, H2A, H2B, H3 and H4 that form an octomer (2 of each core histone) wrapped by 146 bp of DNA^19^. Histones have protruding N-terminal tails that contain lysine residues, which can undergo post-translational modifications by protein complexes containing histone-modifying enzymes^20^. These enzymes include histone acetyltransferases (HATs) that add acetyl groups to lysine residues, a process that leads to recruitment of TFs to gene promoters and facilitation of transcription^21^. In contrast, complexes containing histone deacetylases (HDACs) facilitate the removal of acetyl groups from lysine and recruit repressors that inhibit transcription^22^. Currently, there are four known classes of HDACs that include Class I (HDAC 1, 2, 3, 8), Class II (HDAC 4, 5, 6, 7, 9, 10), Class III (Sirt1–7), and Class IV (HDAC 11)^23^,^24^. The Class I HDACs have received much attention due to their nuclear localization, ability to regulate gene expression, and involvement in drug-induced behaviors^25^,^26^,^27^,^28^. For example, HDAC1 is recruited to the *Fos* gene promoter and regulates its transcription following amphetamine administration^29^. Similarly, intra-NAc infusion of MS-275, a Class I HDAC inhibitor, blocks cocaine-induced locomotor sensitization in mice^30^. An intra-NAc infusion of suberoylanilide hydroxamic acid (SAHA), a Class I and II HDAC inhibitor, was able to enhance cocaine self-administration^31^. Moreover, our group has shown that a single METH injection produced time-dependent alterations in IEG expression that were accompanied by increased nuclear HDAC2 protein accumulation in the rat NAc^16^. However, these data did not clarify whether HDAC2 had any direct or indirect effects on the regulation of METH-induced changes in IEG expression.

Interestingly, psychostimulant-induced increases in IEG expression are followed by a rapid return to normal values^13^,^17^,^32^. The early increases in IEG expression are dependent, in part, on the activation of the CREB signaling pathway via CREB phosphorylation (pCREB)^33^,^34^,^35^. However, less is known about the potential role of other proteins in the regulation of these IEGs. Given that HDAC2-containing complexes can negatively impact gene expression^19^, we sought to determine if loss of HDAC2 would alter the time course of acute METH-induced changes in IEG expression in the NAc. Towards that end, we generated conditional HDAC2-deficient mice using the Cre-loxP recombination system to delete HDAC2 in cells expressing CaMKIIalpha in the brain several weeks after birth. Herein, we compared the acute effects of METH on IEG expression in wild-type (WT) and HDAC2-deficient mice. We found that a single injection of METH (20 mg/kg) triggers time-dependent increases in IEG expression in both genotypes, with some IEGs showing more prolonged changes in HDAC2KO compared to WT mice. Additionally, in WT mice, increased enrichment of HDAC2 on the promoters of several IEGs was observed at a time when the expression of these genes was returning back to normal values. Finally, the prolonged time-dependent increases in IEG expression in HDAC2KO mice were associated with greater pCREB enrichment on the promoters of these genes.

## Results

### Conditional deletion of HDAC2 does not alter baseline IEG expression

**Figure 1:**
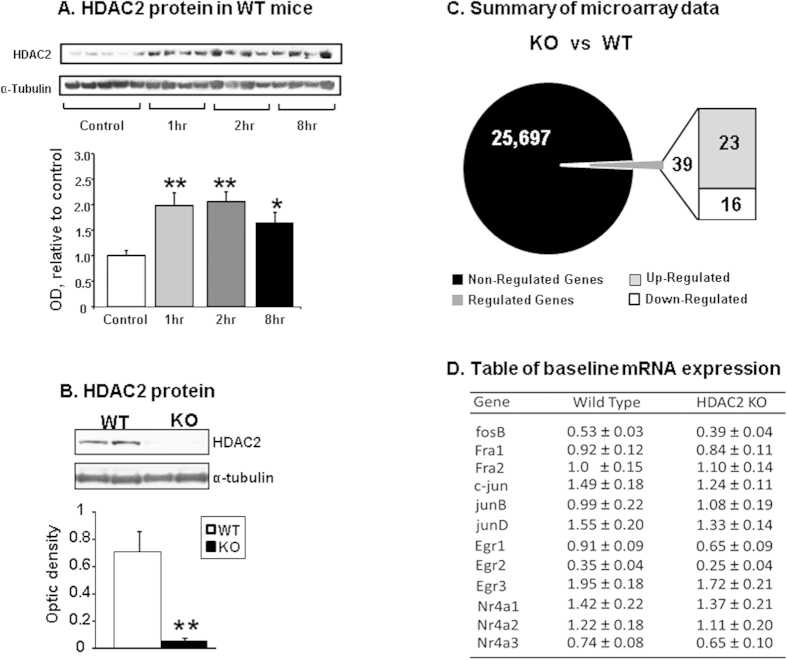
WT and HDAC2KO mice show no differences in baseline IEG mRNA levels. (**A**) METH caused increased HDAC2 protein accumulation in nuclear fractions of NAc from WT mice. The graphs show results from Western blot analyses (N = 6–7 per group) using a specific antibody against HDAC2. The values were normalized to α-Tubulin levels. (**B**) HDAC2KO mice show little HDAC2 protein expression in the NAc relative to WT mice (N = 4 per group) (**C**) Microarray analyses (N = 4 mice per group) revealed that only a few genes were differentially expressed in the NAc of HDAC2 mice. The total number of non-regulated genes represented in black and regulated genes is represented in dark grey in the circle representing the total number of probes. The number of genes with increased expression in HDAC2KO mice is shown in the light grey box and the number of genes with decreased expression is shown in the white box. (**D**) RT-qPCR analyses validated the microarray data showing similar IEG baseline mRNA expression between the two genotypes. The table shows expression levels of IEGs normalized to Clathrin. Values represent means ± SEM (N = 6–8 per group) Key to statistics: Significance was determined by using ANOVA or unpaired Student t test *p < 0.05, **p < 0.01.

The generation of HDAC2 knockout mice is detailed in the method section. Similar to our previous report in rats^16^, an acute METH injection (20 mg/kg) also caused increased HDAC2 protein expression in the NAc of WT mice [*F*(3,18) = 6.72, p < 0.005] (Fig. 1A). Having shown that METH can cause increased HDAC2 expression in mice, we decided to test the effects of HDAC2 deletion on gene expression by using conditional knockout mice in which HDAC2 was deleted in CaMKIIalpha-expressing cells in the brain. Figure 1B shows that there was almost complete disappearance of HDAC2 protein in the HDAC2KO mice [*F*(_1,6_) = 40.94, p < 0.001]. Microarray analysis was employed to measure global gene expression between the two genotypes using MouseRef-8 v2.0 Illumina arrays that contain 25,697 probes. Using a stringent cut-off of 2.0-fold change at p < 0.002, we found that HDCA2 loss was associated with changes in the expression of only 39 genes (23 up-, and 16 down-regulated genes) in comparison to WT mice (Fig. 1C). Supplementary Table 1 shows a partial list of differentially expressed genes between the two genotypes. That list did not include any of the IEGs that are acutely affected by psychostimulants. We also used RT-qPCR to validate the lack of differences in baseline IEG expression in the two genotypes as shown in Fig. 1D.

### Effects of METH on the expression of members of the AP1 family of transcription factors in HDAC2KO mice

**Figure 2:**
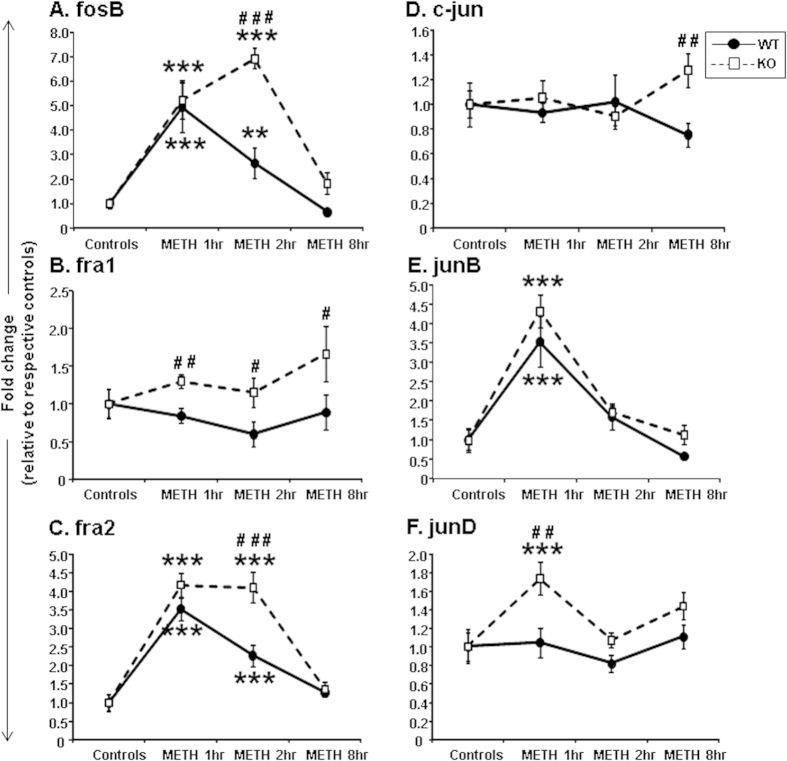
METH caused differential changes in the expression of members of the *Fos* and *Jun* families of IEGs: (**A**) *Fosb*, (**B**) *Fra1*, (**C**) *Fra2*, (**D**) c-*jun*, (**E**) *Junb*, and (**F**) *Jund*. WT and HDAC2KO mice were injected with a single injection of METH (20 mg/kg) and euthanized at 1, 2, and 8 hours after the injection. The relative amounts of transcripts were normalized to Clathrin and expressed as fold-changes in comparison to saline-injected mice of respective genotype. Values represent means ± SEM (N = 6–8 mice per group per time point). Statistical significance between groups was determined by two-way ANOVAs followed by LSD post-hoc tests. Key to statistics: **p < 0.01; ***p < 0.001, in comparison to respective controls of same genotype; ^#^p < 0.05; ^##^p < 0.01; ^###^p < 0.001, in comparison to METH-treated WT mice euthanized at the same time after the METH injection.

Having demonstrated that HDAC2 deletion did not impact the baseline expression of these IEGs, we administered a single injection of METH (20 mg/kg) to measure IEG expression in mice euthanized at various times after drug injection. We observed significant increases in *Fosb* mRNA levels in both genotypes [*F*(_3,52_) = 48.14, p < 0.001] (Fig. 2A). There were also significant genotype*METH interactions [*F*(_3,52_) = 7.77, p < 0.001]. The initial increases in *Fosb* mRNA were comparable in both genotypes at 1 hour after METH (Fig. 2A). However, while HDAC2KO mice showed further increases (6.3-fold) in *Fosb* expression at the 2-hr time point (Fig. 2A), *Fosb* mRNA levels in WT mice were declining towards normal values (2.5-fold), with there being significant differences between genotypes (p < 0.001). These findings were further validated using various doses of METH (1, 5, and 10 mg/kg) in a different set of WT and HDAC2KO mice that were euthanized at the 2-hr time point (see Supplementary Fig. 1A). The effects of METH on fra1 mRNA levels are shown in Fig. 2B. There was an effect of genotype [*F*(_1,51_) = 10.26, p < 0.005], with KO mice having higher *Fra1* mRNA expression at the 3 time points after the METH challenge.

The METH-induced effects on *Fra2* are shown in Fig. 2C. These results are somewhat similar to those observed for *Fosb* expression (compare Fig. 2A–C). There were main effects of METH [*F*(_3,52_) = 73.19, p < 0.001] on *Fra2* expression and significant genotype*METH interactions [*F*(_3,52_) = 6.2, p < 0.001]. Interestingly, as *Fra2* expression was reverting to baseline values in the WT mice (2.2-fold), HDAC2KO mice continued to show higher (4.0-fold) *Fra2* mRNA levels at the 2-hr time point (Fig. 2C). These observations were also validated using different doses of METH using a different set of mice euthanized at the 2-hr time point (see Supplementary Fig. 1B). Figure 2D shows the effects of METH on c-*jun* expression. There were no significant main effects [*F*(_3,52_) = 0.4, p = 0.13] (Fig. 2D). In contrast, METH caused comparable and significant increases in *Junb* expression in both genotypes [*F*(_3,51_) = 38.41, p < 0.001] (Fig. 2E). The levels returned to normal at 2 hours in both genotypes. Figure 2F shows the effects of METH on *Jund* mRNA levels that show significant main effects of genotype [*F*(_1,52_) = 9.85, p < 0.005], with only HDAC2KO mice showing increases at the 1-hr time point.

### Effects of HDAC2 deletion on METH-induced expression of members of the *Egr* and *Nr4a* family of transcription factors

**Figure 3:**
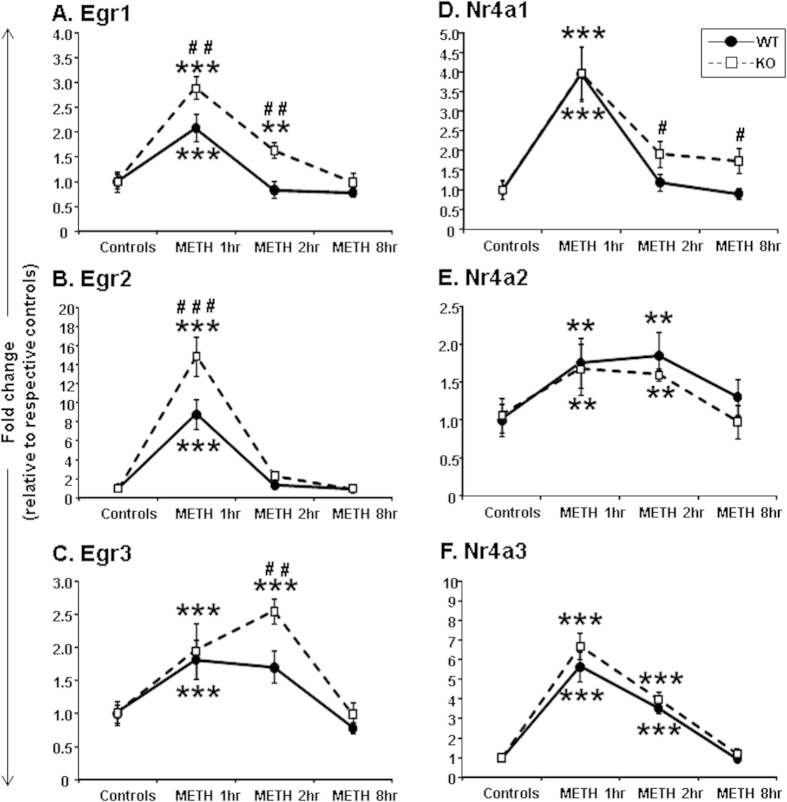
Effects of acute METH treatment on the expression of *Egr* and *Nr4a* family members of IEGs: (**A**) *Egr1*, (**B**) *Egr2*, (**C**) *Egr3*, (**D**) *Nr4a1*, (**E**) *Nr4a2*, and (**F**) *Nr4a3*. METH treatment, RNA extraction, PCR, and statistical analyses are as described in the methods section and in Fig. 2. Data were normalized to Clathrin and expressed as fold-changes in comparison to saline-injected mice. Values represent means ± SEM. Key to statistics: **p < 0.01; ***p < 0.001, in comparison to respective controls of same genotype; ^#^p < 0.05; ^##^p < 0.01; ^###^p < 0.001, in comparison to METH-treated WT mice euthanized at the same time after drug injection.

Previous work in our laboratory had demonstrated that an acute METH injection increased striatal mRNA expression of the *Egr* family members in rats^36^. We thus tested if HDAC2 deletion might also influence METH-induced changes in *Egr* expression (Fig. 3A). METH caused significant increases in *Egr1* mRNA levels in both KO and WT mice [*F*(_3,52_) = 32.47, p < 0.001]. There were also significant genotype*METH interactions [*F*(_3,52_) = 3.1, p < 0.05]. *Egr1* mRNA levels in WT mice returned to normal at 2 hours after the METH injection but were still increased (1.6-fold) in the KO mice (Fig. 3A). Importantly, there were significant differences between genotypes at the 1-hr and 2-hr time points after the METH injection (p < 0.005). The increased expression of *Egr1* mRNA, at the 2-hr time point, in HDAC2KO mice was also confirmed using lower doses of METH (see Supplementary Fig. 1D). METH also caused significant increased *Egr2* expression in both genotypes [*F*(_3,52_) = 90.11, p < 0.001]. There were also significant genotype*treatment interactions [*F*(_3,52_) = 6.8, p < 0.005] (Fig. 3B). *Egr2* mRNA levels were increased by 8.8-fold in WT mice and by 14.8-fold in KO mice at 1 hour after the METH injection (Fig. 3B). The levels of *Egr2* mRNA expression returned to normal at 2 hours in both genotypes. METH caused significant increases in *Egr3* mRNA levels in both KO and WT mice [*F*(_3,52_) = 17.61, p < 0.001] (Fig. 3C). There were also significant genotype*METH interactions [*F*(_3,52_) = 2.88, p < 0.05]. The levels of *Egr3* mRNA were comparable in the two genotypes at the 1-hr time point. However, while *Egr3* mRNA levels in KO mice increased further to 2.5-fold at 2 hours after the METH injection, these values were normalizing in WT mice. Similar findings were also observed using lower METH doses (Supplementary Fig. 1E).

Figure 3 also illustrates the effects of METH on the *Nr4a* family members. METH treatment caused significantly increased *Nr4a1* mRNA levels in both genotypes [*F*(_3,52_) = 30.8, p < 0.001] (Fig. 3D). The initial METH-induced increases in expression were comparable in the two genotypes (3.9-fold for WT and 3.9-fold for KO mice) at 1-hr whereas there were significant differences between the two genotypes at 2 and 8 hours after METH. Specific differences in *Nr4a1* gene expression between the two genotypes at the 2-hr time point were also observed using lower doses of METH (Supplementary Fig. 1F).

*Nr4a2* mRNA levels showed significant effects of METH in both genotypes [*F*(_3,52_) = 5.55, p < 0.005]. The increases in *Nr4a2* mRNA levels were comparable in both genotypes at 1 and 2 hours after METH, returning to normal at the 8-hr time point (Fig. 3E). Figure 3F shows the effects of METH on *Nr4a3* expression. There were main effects of METH in both genotypes [*F*(_3,52_) = 94.7, p < 0.001]. These increases in *Nr4a3* were of greater magnitude than the changes observed for *Nr4a1* and *Nr4a2* mRNA levels (compare Fig. 3D–F). *Nr4a3* mRNA levels were comparable in both genotypes at 1, 2 and 8 hours after the METH injection.

### METH produced increased HDAC2 binding at IEG promoters of WT mice

**Figure 4:**
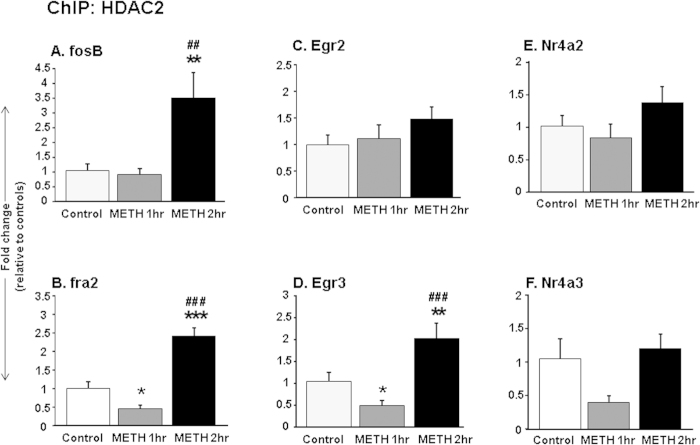
METH-induced increased enrichment of HDAC2 on IEG promoters in WT mice. Chromatin immunoprecipitation (ChIP) assay was used to measure HDAC2 abundance on the promoters of (**A**) *Fosb*, (**B**) *Fra2*, (**C**) *Egr2*, (**D**) *Egr3*, (**E**) *Nr4a2*, and (**F**) *Nr4a3*. Increased HDAC2 binding was observed on *Fosb*, *Fra2* and *Egr3* promoters at 2 hours after METH, corresponding to a time when the mRNA levels of these genes were reverting towards normal values. The relative amounts of HDAC2-immunoprecipitated DNA were normalized to 10% of input control and expressed as fold-changes in comparison to saline-injected mice. Values represent means ± SEM (N = 7–9 per time point). Key to statistics: *p < 0.05, **p < 0.01, significantly different from saline treated WT mice, ^##^p < 0.01; ^###^p < 0.001 in comparison to METH-treated WT mice at the 1-hr time point.

**Figure 5:**
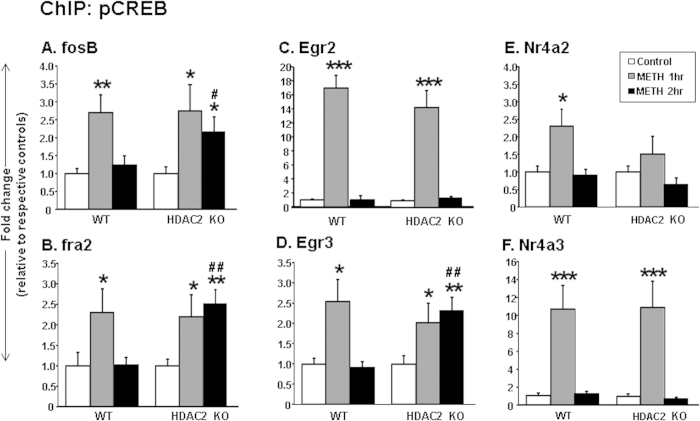
METH produced time-dependent pCREB enrichment on IEG promoters of (**A**) *Fosb*, (**B**) *Fra2*, (**C**) *Egr2*, (**D**) *Egr3*, (**E**) *Nr4a2*, and (**F**) *Nr4a3*. At 2-hrs after the METH injection, pCREB enrichment on the promoters of *Fosb*, *Fra2*, and *Egr3* was increased only in HDAC2KO mice that also showed increased expression of these genes. The relative amounts of pCREB-immunoprecipitated DNA were normalized to 10% of input control and expressed as fold-changes in comparison to saline-injected mice of respective genotype. Data are presented as means ± SEM (N = 7–9 animals per group per time point). Statistical significance between groups was determined by two-way ANOVAs followed by post-hoc tests. Key to statistics: *p < 0.05; **p < 0.01; ***p < 0.001, in comparison to respective saline-treated controls of same genotype; ^#^p < 0.05; ^##^p < 0.01, in comparison to METH-treated WT mice euthanized at the 2-hr time point.

To further test the potential role of HDAC2 in METH-induced changes in IEG expression in the NAc, we measured HDAC2 enrichment on promoters of several IEGs in WT mice at 1 and 2 hours after the METH injection. Figures 4A show the effects of METH on HDAC2 enrichment on the *Fosb* promoter in WT mice. There were main effects of drug treatment [*F*(_2,27_) = 6.2, p < 0.01]. HDAC2 binding on the *Fosb* promoter was increased at the 2-hr time point (p < 0.05), at a time when the levels of *Fosb* mRNA levels were returning towards normal values (see Fig. 2A). The effects of METH on HDAC2 at the promoter of *Fra2* are shown in Fig. 4B. There were also main effects of drug treatment [*F*(_2,25_) = 29.5, p < 0.05]. HDAC2 binding was decreased at the 1-hr time after METH (p < 0.05), at a time when *Fra2* mRNA expression was significantly increased in the WT mice. In contrast, HDAC2 binding to the *Fra2* promoter was increased at the 2-hr time point (p < 0.05) (Fig. 5B) and these increases corresponded to levels of *Fra2* mRNA levels reverting towards normal (see Fig. 2C). Figure 4C shows the effects of METH on HDAC2 at the promoter of *Egr2*. There were no significant main effects [*F*(_2,24_) = 2.4, p = 0.11]. Similar to the observation for *Fra2*, there were main effects of drug treatment [*F*(_2,25_) = 11.25, p < 0.01] on HDAC2 enrichment on the promoter of *Egr3* (Fig. 4D). HDAC2 binding was decreased at the 1-hr time after METH on the promoter of *Egr3* (p < 0.05) when *Egr3* mRNA expression was significantly increased (Fig. 3C). In contrast, HDAC2 binding was significantly increased at the 2-hr time point (p < 0.05) (Fig. 4D) when *Egr3* mRNA levels had started to decline towards normal (Fig. 3C). There were no main effects of METH on HDAC2 enrichment on the *Nr4a2* or *Nr4a3* promoters.

### METH enhanced pCREB binding at IEG promoters of HDAC2KO mice

Because stimulant-induced changes in IEG expression are regulated, in part, by pCREB binding^36^,^37^, we considered the possibility that HDAC2 deletion might also influence pCREB binding on the promoters of the genes that showed increased expression in the HDAC2KO mice at the 2-hr time point. Figure 5A shows the effects of METH on pCREB enrichment on the *Fosb* promoter. There were main effects of drug treatment [F(_2,38_) = 7.9, p < 0.01]. pCREB binding on the *Fosb* promoter was increased in both genotypes at the 1-hr time point. Interestingly, only HDAC2KO showed increased pCREB binding at the 2-hr time point (Fig. 5A). The effects of METH on pCREB at the promoter of *Fra2* are shown in Fig. 5B. There were also main effects of drug treatment [*F*(_2,38_) = 5.1, p < 0.05]. pCREB binding was increased at the 1-hr time after METH in both genotypes, but only the HDAC2KO showed increased binding at the later time. The effects of METH on pCREB enrichment on the *Egr2* promoter are shown in Fig. 5C. There were significant main effects of drug treatment [*F*(_2,35_) = 73.13, p < 0.001], with increased enrichment of pCREB on the *Egr2* promoter at the 1-hr time point being observed in both genotypes. METH caused significant increased enrichment of pCREB on the promoter of *Egr3* in both genotypes, with significant genotype*METH interactions [*F*(_2,35_) = 6.1, p < 0.05] (Fig. 5D). Specifically, pCREB enrichment was significantly increased in both genotypes at the 1-hr time point and was still increased in HDAC2KO, but not in WT, mice (p < 0.05), at the 2-hr time point. There were main effects of METH on pCREB enrichment on the *Nr4a2* promoter [*F*(_2,35_) = 7.9, p < 0.01] (Fig. 5E). Finally, there were significant main effects of METH [*F*(_2,34_) = 18.13, p < 0.001] on pCREB binding on the promoter of *Nr4a3* at the 1-hr time point in both genotypes. The observations of increased pCREB binding on the promoters of *Fosb*, *Fra2*, and *Egr3* in the HDAC2KO mice at the 2-hr time point are consistent with the findings that these genes showed prolonged mRNA expression in comparison to WT mice.

## Discussion

The NAc is a brain structure that has been implicated in addiction^12^. Because psychostimulant-induced changes in some IEGs may contribute to the molecular adaptations that subsume addiction^7^,^38^, several groups of investigators have sought to understand the impact of various licit and illicit substances on their expression and regulation in the brain^13^,^39^. It has been shown, for example, that several psychostimulants regulate IEG expression via increased pCREB binding at their promoters in models of acute drug administration or drug self-administration^8^,^33^,^34^,^35^. Nevertheless, much remains to be done to further clarify the epigenetic bases for these drug-induced changes in IEG expression. For example, the induction observed after acute stimulant injections is usually short-lived, suggesting the possibility that molecular mechanisms that involve repressor complexes might be involved in the regulation of the expression of these genes. In the present study, we assessed the potential role of HDAC2 in regulating the time course of IEG expression by comparing responses in WT and HDAC2KO mice. Our main findings are that: (1) METH caused early increases in the expression of several IEGs including members of AP1, Egr, and Nr4a TF families in the NAc of both WT and HDAC2KO mice; (2) METH-induced increased *Fosb*, *Fra2*, and *Egr3* expression was more prolonged in the HDAC2KO mice in comparison to WT mice; (3) METH also caused increased abundance of HDAC2 on the promoters of *Fosb*, *Fra2*, and *Egr3* in WT mice; and (3) HDAC2KO mice showed prolonged increased pCREB binding on the promoters of *Fosb*, *Fra2*, and *Egr3*.

The effects of an acute METH injection include the induction of several IEG and transcription factors in various brain regions^14^,^15^,^16^,^17^,^39^. The present study confirms these results and extends this literature by showing that *Fosb* and *Fra2* mRNA levels remained elevated in HDAC2KO mice while these values were returning to normal in WT mice, as reported by previous studies^13^,^16^. The present observations are consistent with a report demonstrating that mice with NAc-specific deletion of HDAC3, another member of the Class I HDACs, showed higher cocaine-induced *fos* mRNA expression in comparison to WT mice^40^. These observations are also consistent with the fact that pre-treatment with SAHA, a HDAC inhibitor, potentiated cocaine-induced increases in *Fosb* mRNA levels^41^. The report that RGFP966, a selective HDAC3 inhibitor, inhibited HDAC-induced suppression of IEG expression after cocaine treatment^42^ also corroborates our findings. This discussion is relevant to our experiments investigating the possibility that METH might alter HDAC2 abundance on the promoters of some IEGs, including those that showed increased differential expression in the two genotypes. Indeed, we found that METH caused decreased HDAC2 binding on the promoters of *Fra2* and *Egr3* in WT mice at 1-hr after the drug injection, at a time that both genes also showed METH-induced increased mRNA expression in the WT mice. These observations suggest the possibility that decreased HDAC2 binding might have led to increased histone acetylation with consequent expression of these genes. This idea is supported by previous observations that decreased HDAC2 activity is associated with increased histone acetylation in the rodent brain^43^. This suggestion is also consistent with the increased HDAC2 binding on the promoters of genes whose expression was beginning to normalize at the 2-hr time point in WT mice. Thus, our epigenetic data support the hypothesis that HDAC2 is a negative regular of METH-induced increased expression of some IEGs in the brain.

Although our observations supported a role for HDAC2 in the regulation of some of these IEGs, it was important to examine the participation of pCREB recruitment on IEG promoters since pCREB is a known mediator of METH-induced IEG expression^8^,^33^,^34^,^35^. This was of particular interest because HDAC inhibition has been shown to modulate memory function in the hippocampus via CREB/CBP-mediated gene expression^44^,^45^. Indeed, we found increased pCREB abundance on the promoters of genes that showed higher METH-induced expression in HDAC2KO mice at the 2-hr time point. Although these observations provide a correlation between changes in pCREB binding and the prolonged METH-induced IEG expression in the absence of HDAC2, additional studies are required to demonstrate a causal relationship between lack of HDAC2, changes in pCREB binding, and METH-induced increases in IEG expression. This is of particular importance because CREB phosphorylation is known to lead to the recruitment of the acetyl-transferase, CREB-binding protein (CBP)^46^,^47^ to gene promoters. This is followed by CBP-induced histone^48^,^49^ and CREB^50^ acetylation that enhances CREB-mediated gene expression. Thus, the lack of HDAC2 might have increased the recruitment of CBP to gene promoters, resulting in hyperacetylation and prolonged pCREB binding on the promoters of the METH-regulated IEGs. This argument is supported by the fact that the HDAC inhibitor, sodium butyrate, increases pCREB enrichment on promoters of neuronal plasticity related genes in the hippocampus^51^. Our conclusion is also consistent with the demonstration that, in the absence of HDAC2, increased pCREB binding allows the transcription of latent TGFbeta-binding protein-1 (Ltbp1), a regulator of TGFbeta activation^52^.

It is also tempting to speculate on the specificity of HDACs in their regulation of CREB targets genes, giving our findings that several of the genes with METH-induced gene expression contain CREB binding sites. However, previous reports have indicated that deletions of HDAC2^43^ and HDAC3^40^ can also alter the expression of genes that are not specific pCREB targets. Also of note is the possibility that some of the genes regulated by HDAC2 might be targets of serum response factor (SRF)^53^ and of myocyte enhancer factor 2 (MEF2)^54^ because cocaine can regulate gene expression via activation of these transcription factors^35^,^55^. This is indeed a possibility since HDAC4 has been shown to interact with MEF2 and SRF to mediate the suppression of their target genes^56^,^57^. This discussion thus suggests that more experiments are needed to completely elucidate the role of HDACs in the regulation of gene expression after administration of psychostimulants.

In conclusion, a single METH injection induces significant increases in IEG expression in the mouse NAc. In addition, we report, for the first time, that HDAC2 may be involved in the acute transcriptional responses to METH exposure. These observations are consistent with previous research showing that histone-modifying enzymes play a central role in the regulation of psychostimulant-induced changes in gene expression^28^. The fact that manipulations of these enzymes can also alter drug-induced behaviors in rodents^42^ also supports this suggestion. Our findings also suggest that, in the absence of HDAC2, prolonged induction of some IEGs is associated with prolonged activation of the adenylate cyclase/cAMP/PKA/CREB pathway. Because METH exposure can cause alterations in epigenetic mechanisms in the brain^28^,^58^, it is possible that these early effects might drive long-lasting METH-mediated behaviors. Finally, our study provides further support for the accumulating literature indicating that HDACs might be important targets for therapeutic interventions in METH addiction.

## Methods

### Animals

An initial cohort of HDAC2/loxP transgenic mice was obtained from Charles River Laboratories (Frederick, MD, through the generosity of Eric Nestler, Mount Sinai Hospital) and c57BL/6 CaMKIIalpha Cre transgenic mice were purchased from the Jackson’s Lab (Bar Harbor, ME). The mice were then crossed in the breeding facility of the National Institute of Drug Abuse (NIDA) Biomedical Research Center (BRC) in Baltimore, MD. Two hundred and forty two (242) male mice were genotyped by Charles River’s Laboratory Testing Management® (LTM) division and used in the experiments.

### Drug treatment

Male mice (12–14 weeks old, weighing 30–35 g) received a single intraperitoneal (ip) injection of saline or METH (20 mg/kg) and were euthanized at various time points after the injection (1-hr, 2-hrs or 8-hrs). The METH dose and time course were based on previous experiments in which we observed increased HDAC2 protein expression in the rat NAc^16^. All animal procedures in this experiment were conducted according to the NIH Guide for the Care and Use of Laboratory Animals and were approved by the NIDA-/Intramural Research Program (IRP) Animal Care and Use Committee (NIDA/IRP-ACUC).

### RNA extraction and RT-qPCR

The NAc was dissected, immediately placed on dry ice and stored at −80 °C. Total RNA was isolated using the Qiagen RNeasy® Mini kit (Qiagen, Valencia, CA). Analysis of RNA integrity was assessed using an Agilent 2100 Bioanalyzer (Agilent, Palo Alto, CA). Total RNA was then reverse-transcribed to cDNA using oligo dT primer from the Advantage RT for PCR kit (Clontech, Mountain View, CA). Gene-specific primers were designed using the LightCycler Probe Design software version 1 (Roche, Indianapolis, IN) and purchased from the Synthesis and Sequencing Facility of Johns Hopkins University (Baltimore, MD). The specific primers used can be obtained from the authors upon request. IEG expression levels were examined using a LightCycler 480 II (Roche). The normalization of qPCR values was performed using clathrin (*Cltc)* as the reference gene.

### Microarray hybridization

We used MouseRef-8 BeadChips arrays (25,697 probes) (Illumina Inc., San Diego, CA) for microarray analysis. Briefly, raw data were imported into GeneSpring software v.12 (Agilent) and normalized by global normalization. The normalized data were then used to identify changes in gene expression. Individual genes were identified as having significant increased or decreased expression based on an arbitrary cut-off of 2.0-fold change at p < 0.002, using unpaired t-tests according to the GeneSpring statistical package. We have used similar criteria effectively for our other publications^14^. Data are shown as fold-changes calculated as the ratios of normalized gene expression between HDAC2KO and WT mice.

### Chromatin Immunoprecipitation (ChIP)-qPCR

Briefly, minced tissue was cross-linked in 1% formaldehyde/PBS for 15 minutes and stopped by the addition of glycine (0.125 M) as described previously^8^,^27^. Dynabeads (Life Technologies, Grand Island, NY) were incubated with 5 μg of antibodies raised against HDAC2 (ab12169) (Abcam, Cambridge, MA) or phospho-CREB (9191L) (Cell Signaling, Danvers, MA) overnight at 4 °C. Equal amounts of chromatin lysate (50 μg) were diluted with ChIP dilution buffer (Millipore, Billerica, MA) and immunoprecipitation was carried out overnight at 4 °C. For normalization, 10% of chromatin pre-immunoprecipitated lysate was used as “input control”. DNA-protein complexes were then disassociated at 65 °C and treated with RNase A and proteinase K (Life Technologies). DNA was then isolated using phenol/chloroform extractions and suspended in 10 mM Tris pH 8.0. PCR was performed on ChIP-derived DNA using a LightCycler 480 II (Roche). PCR reactions were performed in duplicate for each gene and threshold amplification cycle numbers (Tc) were used to calculate DNA quantities.

### Western blot analysis

Briefly, frozen samples were homogenized in ice-cold buffer as described previously^8^,^16^. Nuclear fractions were suspended in buffer containing 20 mM HEPES, 840 mM NaCl, 0.5 mM MgCl2, 4 mM EDTA, 10% glycerol, protease and phosphatase inhibitor cocktail tablets (Roche). Protein concentrations from nuclear fractions were determined by utilizing the BCA assay kit (Thermo Fisher Scientific, Rockford, IL). Samples were then electrophoretically transferred on to PVDF membranes and incubated overnight at 4 °C with a specific antibody against HDAC2 (1:1000) (Cell Signaling). Membranes were then re-probed with an antibody against α-Tubulin (1:6000) (Sigma, St. Louis, MO). Protein signal intensity was measured on the with the Kodak image station 4000 pro (Kodak, Rochester, NY) using the Carestream Molecular Imaging software.

### Statistical Analyses

Microarray data were analyzed using unpaired t-tests (GeneSpring, Agilent Technologies, Savage, MD. Quantitative-PCR, ChIP-PCR and western blot data were analyzed using two-way ANOVAs followed by Fisher’s LSD where appropriate (SPSS 20, IBM, Armonk, NY). All data are presented as means ± SEM and considered statistically significant when p ≤ 0.05.

## Additional Information

**How to cite this article**: Torres, O. V. *et al*. CAMKII-conditional deletion of histone deacetylase 2 potentiates acute methamphetamine-induced expression of immediate early genes in the mouse nucleus accumbens. *Sci. Rep*. **5**, 13396; doi: 10.1038/srep13396 (2015).

## Supplementary Material

Supplementary Information
