# Supplementary material for: CAMKII-conditional deletion of histone deacetylase 2 potentiates acute methamphetamine-induced expression of immediate early genes in the mouse nucleus accumbens
Source: Sci Rep. 2015 Aug 24;5:13396. doi: 10.1038/srep13396 (PMC4547138; doi:10.1038/srep13396)
Supplement: Supplementary Information [file srep13396-s1.pdf]

CAMKII-conditional deletion of histone deacetylase 2 potentiates acute methamphetamine-induced expression of immediate early genes in the mouse nucleus accumbens

Oscar V. Torres, Ph.D. [1], Michael T. McCoy, M.S. [1], Bruce Ladenheim, B.S. [1], Subramanian Jayanthi, Ph.D. [1], Christie Brannock, MBA[1], Ingrid Tulloch, Ph.D. [2], Irina N. Krasnova, Ph.D. [1], and Jean Lud Cadet, M.D. [1]*

[1]Molecular Neuropsychiatry Research Branch, DHHS/NIH/NIDA Intramural Research Program, 251 Bayview Boulevard, Baltimore, MD 21224 and [2]Department of Psychology, Stevenson University, Stevenson, MD 21283.

Symbol	Fold change	Definition	Function
Tcam1	8.64	testicular cell adhesion molecule 1	encodes testis-specific cell adhesion proteins for germ cell interactions
Olfr571	8.63	olfactory receptor 571	recognition of odorant signals by G protein-mediated transduction
Nek11	8.62	never in mitosis gene related kinase 11	DNA replication and response to genotoxic stress
Rfx4	7.79	regulatory factor X, 4	influences HLA class II expression
Thbd	7.75	thrombomodulin	endothelial-specific type I membrane receptor that binds thrombin
Pde6g	6.82	phosphodiesterase 6G	regulates G-protein-coupled receptor kinase 2
Mtnr1a	6.06	melatonin receptor 1A	G-protein coupled melatonin receptor involved in circadian rhythm
Csf3r	5.54	colony stimulating factor 3 receptor	controls production and function of granulocytes
Angptl4	2.09	angiopoietin-like 4	regulation of glucose homeostasis, lipid metabolism, and insulin sensitivity
Cnn2	2.03	calponin 2	regulation and modulation of smooth muscle contraction
Olfr800	-2.90	olfactory receptor 800	interact with odorant molecules to initiate the perception of a smell
Dip2b	-3.21	DIP2 disco-interacting protein 2 homolog B	encoded protein for DNA methyltransferase binding site
Rab19	-3.58	member RAS oncogene family 19	important for intracellular transport
C3ar1	-3.64	complement component 3a receptor 1	receptor for the inflammatory peptide anaphylatoxin C3a
Wnt2b	-3.76	wingless related MMTV integration site 2b	signaling factors for the regulation of cell growth and differentiation
Olfr192	-3.78	olfactory receptor 192	interact with odorant molecules to initiate the perception of a smell
Lamc2	-4.82	laminin, gamma 2	extracellular glycoprotein involved in cell adhesion, migration, and signaling
Rin2	-4.92	Ras and Rab interactor 2	functions as a guanine nucleotide exchange factor for the RAB5 protein
Tnpo1	-7.60	transportin 1, transcript variant 1	formation of karyopherin receptors for localization of nuclear proteins

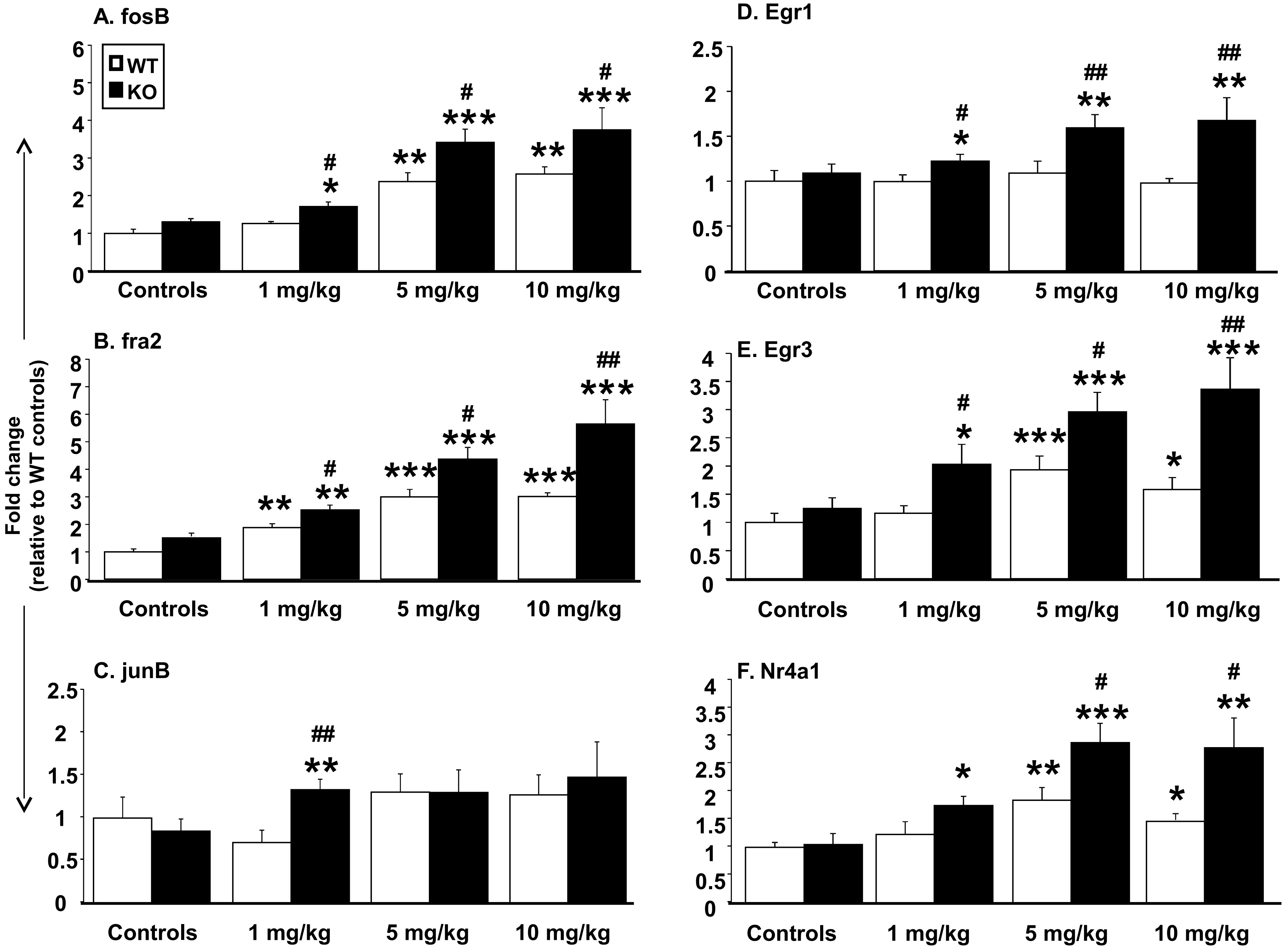
A. fosB
B. fra2
C. junB
D. Egr1
E. Egr3
F. Nr4a1
WT
KO
Fold change (relative to WT controls)
Controls
1 mg/kg
5 mg/kg
10 mg/kg

Supplementary Table 1

List of 39 genes that were altered by deletion of neuronal HDAC2 in the mouse NAc. The table shows up- and down-regulated genes in HDAC2KO mice relative to WT mice as assessed by MouseRef-8 BeadChips arrays with 25,697 gene probes. Moreover, the table shows that HDCA2 loss was not associated with changes in the expression IEGs of interest. There were 4 animals per group for the microarray experiments.

Supplementary Figure 1

Lower doses of METH caused differences in IEG expression between WT and HDAC2KO mice. Separate groups of WT an HDAC2KO mice were injected with a single dose of METH (1, 5, or 10 mg/kg) or saline and sacrificed 2 hours later. Quantitative PCR shows increased METH-induced expression of (A) *Fosb*, (B) *Fra2*, (D) *Egr1*, (E) *Egr3* and (D) *Nr4a1* in NAc of HDAC2KO mice relative to WT mice. These results are similar to our observations with a moderate dose of METH (20 mg/kg), and further validate the METH-induced higher expression of these genes in HDAC2KO mice at the 2 hour time point. The relative amounts of transcripts were normalized to *Oaz1* and expressed as fold-changes in comparison to WT saline-injected mice. The data are represented as means ± SEM (N = 10 mice per group per METH dose). Statistical analyses were performed using paired t-test for each separate dose relative to controls or between respective genotypes. Key to statistics; * = $p<0.05$; ** = $p<0.01$; *** = $p<0.001$, in comparison to respective controls; # = $p<0.05$; ## = $p<0.01$, in comparison to respective METH-treated WT mice.
